# 

**Published:** 1887-04-23

**Authors:** 


The Hospital.

An Institution, Family, and Parochial Journal of Hospitals, Asylums, and all Agencies for the Care of the Sick, Criticism and News.

SATURDAY, APRIL 23RD, 1887.

### Books, Good and Otherwise.

We have received the following for review :—

Asylum for the Deaf and Dumb, 93, Cannon Street.
Deaf and Dumb-land. By Joseph Hatton.
Churchill, J. & A.
The Bristol Medico-Chirurgical Journal for March, 1887.
Longmans & Co.
She. By H. Rider Haggard.
He.
Marrying and Giving in Marriage. By Mrs. Molesworth.
Story of Our Lord. By F. Younghusband.

EXCHANGES.

The Therapeutic Gazette, Detroit, U.S.A, Nos. 1 and 2.
The Medical Record for 26th Feb., 1887.
The Journal of Mental Science, April, 1887.

### Covers for Binding

Vol. I. of THE HOSPITAL may be had of the Publishers, Messrs. Whiting & Co., Sardinia Street, Lincoln's Inn Fields, and of all Booksellers. Price 1*s*. 6*d*. each.

Country Subscribers are requested to order the Covers through their Booksellers, as they cannot be forwarded by post at book rate.

### Hospital Housekeeping.

Forms and full particulars of the Quilter Prizes under this head (see page 355 for 19th Feb.) may be had on application to the Editor of The Hospital, Norfolk House, W.C. All competitive essays to be sent in by 30th April, 1887. Prizes, £10 10*s*. and £5 5*s*.

*** The Children's Corner and Amusements with Profit will be found on page vi of this week's issue.

### Questions and Answers.

All questions should be addressed to Mr. Howard J. Collins, Secretary, The Hospitals Association, Norfolk House, Norfolk Street, Strand, W.C.

### WANTED, A HOUSE SUITABLE FOR A CONVALESCENT HOME.

Mr. H. Thornhill Roxby, Secretary of the After-Care Association for Poor and Friendless Female Convalescents on leaving Asylums for the Insane, writes:—"Mrs. Rusher's Home at Dover cannot be given over for our work. I shall be glad if you can let me know of any other convalescent homes about to be given up, and which might be likely to suit us."

### ONE-YEAR PROBATIONERS.

Miss E. S. B. Landells, Superintendent, Trained Nurses' Home and Institute, Bath, writes:—"Will you kindly inform me if there are any London hospitals, except University College Hospital, which will receive probationer nurses without payment or fees for one year?"

⁂ Does University College do this? If so, it is believed to be the exception which proves the rule.—Ed. T.H.

### THE MUSEUM AT THE ROYAL COLLEGE OF SURGEONS.

"Olive" writes from Ashmount Road, Hornsey Lane, N.:—"I have been for some time trying to ascertain whether the Museum of the Royal College of Surgeons is open to any but members of the medical profession, and if so, by what means I could gain admission for myself and a friend (a private nurse), and seeing a second paper on the Museum in this week's issue of The Hospital, I thought, perhaps, you could give me the information."

⁂ The Museum of the Royal College of Surgeons, Lincoln's Inn Fields, is open on Monday, Tuesday Wednesday, and Thursday, from 11 till 4, to Fellows and Members of the College, or to strangers introduced by them, either personally or by written order. Any member of a learned society is also admitted. The Conservator, Professor Fowler, is usually very courteously inclined to admit any applicant for admission who can show any special reason for the application, but those who are not members of this or any other learned body should provide themselves with written orders from a Fellow or Member of the Royal College of Surgeons, London.—Ed. T. H.

### VARNISHED PAPER FOR HOSPITAL WALLS.

Mr. Henry Parker writes from Potter's Bar:—"On page 454 of Mr. Burdett's book on 'Cottage Hospitals,' J. and A. Churchill, London, he strongly recommends the walls being papered and varnished. We are doing up our hospital, and our managers would like to do as he recommends, but are fearful of too much glare from the varnish, as some of our rooms are very sunny. Can you give me the name of some hospital, in or near London, which you know has been thus papered, so that I could go and look at it?"

⁂ If Mr. Parker will apply to Mrs. Bluett, the very able Superintendent of the Home Hospital, Fitzroy House, Fitzroy Square, London, W., she will, no doubt, be happy to show him a room or rooms with varnished paper where very serious surgical cases have been successfully treated during the last few years. He will thus be able to convince himself that there is no such objection as he seems to fear, but that the general effect is as pleasing as the hygienic effect is satisfactory.—Ed. T. H.

### THE CHILDREN'S CORNER.

**Third Quarterly Prize Competition.**
Began 2nd Apr., 1887 ; ends 25th June, 1887.

PRIZES.

FOR MOST CORRECT ANSWERS TO PUZZLES.

| | | | |
|---|---|---|---|
| 1st .. .. | Thirty Shillings | 3rd .. .. | Ten Shillings |
| 2nd .. .. | Twenty „ | 4th .. .. | Five „ |

FOR NEATEST ANSWERS TO PUZZLES.
Five Shillings.

FOR BEST LITTLE LETTERS.
Ten Shillings.

**Puzzles—Fourth Week.**

No. 15. DECAPITATION.

Cut off my head, the singular I act ;
Cut off my tail, the plural doth appear ;
Cut off both head and tail, astounding fact,
Tho' my middle's left, there is nothing there.
What is my *head !* it is a sounding sea ;
What is my *tail !* it is a bounding stream ;
My *middle's* void, naught really it may be ;
My *whole's* a fish, one very fine you'll deem.

No. 16. BIRDS' NAMES ENIGMATICALLY EXPRESSED. To pass down the throat ; a famous nurse ; a celebrated architect ; a luminous body in the heavens and a fish ; the firmament and fun.

No. 17. ENIGMA.

Cato and Chloe combin'd well together
Make a drink not amiss in very cold weather

No. 18. BURIED WORDS.

In Ethel, not in Ellie ;
In Dot but not in Tim ;
In Nordisa, not in Gom ;
In Peeler and P. M. ;
In Mikado, not in Skye ;
In Clytie, not in A. C. K. ;
In Agatha but not in Scrooge ;
In Alsie and in Isobel.

Find a word of eight letters.

No. 19. CONUNDRUM. Why has Mr. Gladstone never been able to insure his life ?

**Letters—Fourth Week.**

The Letter-subject for next week will be "Flowers

**Results of Second Week—Puzzles.**

No. 6. CONUNDRUM. An oyster is a paradox because—
He wears a beard without a chin,
And leaves his bed to be tuck'd in.

No. 7. BURIED AUTHORS. Campbell, Milton, Swift, Addison.

No. 8. SUBTRACTION.

9, 8, 7, 6, 5, 4, 3, 2, 1=45
1, 2, 3, 4, 5, 6, 7, 8, 9=45

8, 6, 4, 1, 9, 7, 5, 3, 2=45

No. 9. ENIGMA. Gladstone.

*All right*—Dot, R. Epton, Joe, Nordisa ; *Three right*—A. C. K., Isobel, Scrooge, Skye, Gom, Tim, Umslopogaas ; *Two right*—Agatha, Bohemian Girl, Ellie, Mikado, Alsie, Sutton, Peeler ; *One right*—A. G. S. McCulloch.

**Letters.**

The letters last week about "Home" were, I think, hardly up to the average. Some of my little friends clearly did not quite understand my wishes, for they have sent me descriptions of their drawing-rooms and parlours ! "Home" is a very beautiful English word, so expressive and so full of meaning, a word which has no real synonym in French and many other languages. Here is a little note from Ellie about her home :—

DEAR MR. EDITOR,—Our home is a very happy one ; we are as merry as crickets all the day long, although we have not been so much so the last fortnight, on account of my youngest brother Herbert being very ill, but I am thankful to say he is getting well again. Although he is only five years old, he already begins to enjoy the fun we have with THE HOSPITAL ; in fact, he suggested that I should write and tell you that he was ill. My mamma and papa were very pleased that I have been awarded the Prize for Neatness.

Yours faithfully, ELLIE, aged 11.

44, Burgoyne Road, Brixton, S.W.

Here is an entertaining letter from Skye :—

DEAR MR. EDITOR,—Your order for this week is "Home." Well, they say there is "no place like home." I do not know, as I have never been long enough away from home to be able to echo this. I believe the poor man who made this beautiful song, had no home of his own in his later years. My home has been mostly in the country, and a good part of it has been spent in the Island of Skye. I liked it better than any other place I have been in. The sea came up close to the house, and I used to fish for sprats and catch them as fast as I could. This was varied by going for long drives of ten miles sometimes, with papa to visit his patients. Then home *was* nice to come to when hungry and tired,—anything and everything tasted nice. I am sorry to say we have lived since in the town. I do not care so much for it as the country, still one's home is where one's friends are. I suppose it is very much what we make it ourselves. A great deal could be said about this pretty word, but I must not take up more space.

I remain, yours faithfully, SKYE, aged 14.

One mark each :—Bohemian Girl, Ellie, Mikado, Peeler, Skye, Tim.

**Notices to Correspondents.**

SUTTON.—Competitions for Children's Corner must be sent by Thursdays.

R. EPSON.—Please give full Christian name.

**Rules.**

Only one side of the paper should be written upon.

The address and full Christian name and surname, and the age of the competitor, must be stated in all replies. A *nom de plume* may be added for the purposes of publication.

The first prize may not be taken by the same competitor more than once in any one year. In the event of the winner of the first prize again scoring highest, the second prize will be awarded, the next competitor receiving the first.

Answers must be addressed to the "PUZZLE EDITOR," THE HOSPITAL, Norfolk House, Norfolk Street, Strand, W.C., not later than Thursday next. The "Children's Corner" Coupon (see advertisement pages) must be enclosed.

### AMUSEMENTS WITH PROFIT.

### PRIZE COMPETITIONS.

**Quarterly Prize Competition.**
Began 23rd April ; ends 16th July.

**A Prize of Three Guineas**

will be awarded to the Competitor who shall have sent in the largest number of correct or best answers. No one will be permitted to win the Three Guinea Prize twice in any one year, but we shall award annually an additional

**Prize of Five Guineas**

to the competitor who shall have sent in the largest number of correct or best answers during the twelve months.

**No. 1. Double Acrostic.**

An institution in this great city
On Italy's poor sick sons has pity.

This river o'er the lowlands of Siberia flows ;
The site of our first factory in the Mogul's land ;
A cheerless place, by Chili claim'd and Argentine ;
In Donegal this famous mountain peak doth stand ;
A town, the "Rome of India," from its grandeur call'd ;
A wee republic in a forest valley lies ;
'Tis the most ancient town in all Dutch Rhineland found ;
A name for Spain this river's classic name supplies.

**No. 2.—Mathematical Question.**

A mouse climbs up a pole. It gets up three feet a day, but slips back two feet each night. The pole is twenty feet high How many days will it take the mouse to reach the top ?

**Answers.**

No. 23. DOUBLE ACROSTIC.

| | | |
|---|---|---|
| H | ambur | G |
| E | yla | U |
| L | ombard | Y |
| P | oitier | S |

Correct answers have been received from Mater, A. M. E., Mr. J. High, Gip, Boss, Ellice, K. M., Perseverance.

A. M. E. thinks Guy's is a "little hard" on other hospitals in wanting £100.000, but adds, "to show that I take in your kind wish in putting the acrostic, I will send you 1s. if other solvers will do the same for Guy's." What do our competitors say ?

**No. 24. Mathematical Question.**

Let $x$=number of hospitals

$$\text{then } x(x-2)=120$$
$$\text{or } x^2-2x=120$$
$$(x-12)(x+10)=0$$
$$\therefore\quad x=12\text{—Answer.}$$

Correct answers have been received from Gretta, K. M., Gip, Peeler, Mr. J. High, A. M. E., Ellice, Mater, Ostrich, Perseverance, Hexameter.

Answers must be addressed to THE PRIZE EDITOR, AMUSEMENTS WITH PROFIT, THE HOSPITAL, Norfolk House, Norfolk Street, Strand, W.C., not later than the *first* post on Saturday next. The full name and address must in all cases be given, but a *nom de plume* may be added for publication. Only one side of the paper should be written on. The "Amusements with Profit" Coupon (see advertisement pages) must be enclosed with replies.

